# Treatment of Tuberculosis of the Larynx

**Published:** 1887-10

**Authors:** Lennox Browne

**Affiliations:** London


### *RECENT VIEWS AS TO PATHOLOGY AND TREATMENT OF TUBERCULOSIS OF THE LARYNX.*

By Mr. LENNOX BROWNE, of London.

The tubercular bacilli are generally admitted as a cause of specific laryngitis. Entrance is effected through the air passages, and they are especially liable to accumulate in the upper parts of the lungs, while there is less respiratory action. The disease is usually secondary to pulmonary tuberculosis, and may be due to infection from the bacilli in the sputa coughed up, and inoculating some abraded or unhealthy and irritated portion of the larynx, or the germs may find their way thither through the lymphatic system. The bacilli, like all parasites, act as an irritant, and eventually cause breaking down of the tubercular deposits.

They must have an unhealthy or abraded surface on which to locate and thrive, and are prone to choose some weak points. Whether carried by the air, sputa, lymphatics, or general circulation, cases are recorded of infection through wounds, and teeth that have been extracted. The author of the paper quoted cases confirming the same.

The state of the health and the assimilation of food, together with tissue nutrition, have more to do with the development of tuberculosis than have locality or climatic conditions. Not only may we have laryngeal tuberculosis as a secondary infection, but we find cases where it is primary. The laryngeal symptoms may be the first to attract attention, and even become far advanced before pulmonary lesions can be detected. This is confirmed by many observers. The bacilli are recognized in the sputa; there is pain in the larynx and difficult deglutition.

The larynx presents a specific appearance. We find the infiltration, dwelling and ulcers, but still can detect no pulmonary lesion. There must have been some neglected chronic laryngitis, or some solution of continuity when exposed to these germs. Cases are reported where these local symptoms have yielded to treatment before pulmonary complications were added, and the patients were discharged apparently cured.

*Treatment.*—Where the general system is not broken down, or the disease advanced to the lungs, sea or mountain air and high altitudes, especially in pine regions, are of vital importance. Oxygen, pure air and the absence of germs are here more to be relied on than the thermal or climatic agents. Dr. Moore has ceased to advance the inhalation of medicated steam, but prefers oxidizing agents. Inhalations of vapor from turpentine, oil ot eucalyptus and menthol he has used with success.

It is well established that tuberculosis is a blood-poison, as is pyæmia, septicæmia and the like. Germicides should be used, and benefit will result. He places great confidence in atropine, not only as a sedative, but a germicide as well. Arsenic often works in the same manner as does mercury in syphilis. So, also, do the salts of calcium, where there are tubercular deposits. The aniline treatment has not been a success, and the gaseous injection is still on trial. Experiments with sulphuretted hydrogen show temporary improvement, with diminution of the amount of sputa expectorated, less pain and less distress from persistent cough. Still the permanent benefit is doubtful. This treatment needs careful supervision, and should not be trusted to the patients or their friends. The local treatment of tubercular laryngitis gives best results, especially where systemic treatment is also employed to maintain health and nutrition. Use cocaine and employ galvano-cautery or lactic acid to destroy the deposits and induce healthy healing of the parts.

Where the infections are local, our success is in proportion to their accessibility.

Does not like iodoform or iodol dissolved in ether, as the ether is too much of an irritant; prefers a brush made with cotton to the spray, in that it coats the surface better and is pressed into folds which are protected from the spray by the spasm of the larynx. The continued use of the spray he considers dangerous to the ciliæ of the epithelial cells. Local and systemic sedatives are emphatically called for. Insufflation is not as good as where emulsions are made with acacia, as they are apt to form cakes. Cocaine gives temporary relief, but morphia, belladonna and balsam are more permanent in their relief of both pain and cough.

The surgical measures are to scrape away the deposits with curette or forceps, under cocaine, and apply lactic acid. Do not stab or incise; it may relieve tension and congestion, but is bad in that it gives new foci for infection. For the same reason, it is better not to remove gromulerata unless respiration is seriously interfered with. Tracheotomy, for the purpose of giving rest to the larynx, is useless and worse. The larynx does not then receive the necessary air and oxygen, and bacilli-infected mucus accumulates. The cold and dry air irritates the lungs and may induce pulmonary complications. Besides, the wound may become infected. Intubation of the larynx likewise causes too much irritation and aggravates the trouble, independent of the risk of blocking up the tube; even refrain from removing elongated uvula; does not approve of recent suggestion of extirpating diseased portions.

Many apparent cures are reported, and he has had such cases himself, but is inclined to doubt their permanence; can improve condition of larynx, stop pain and cough, cause better assimilation of food, and improve nutrition. There result local reliet and apparently satisfactory results, but scarcely a cure, as claimed by Schmidt, Bosworth and others.

*Important Suggestions.*—(1) Early diagnosis and treatment. (2) Do not be carried away with new remedies. (3) Never be too active in destroying tissue; heal instead. (4) It is better to observe facts and be influenced by the experience of many than the ideas of the few.

Dr. Sinclair Coghill, Ventnor, England, advised to treat more to alleviate pain and distress, which tend to shorten life, and obtain as much rest for parts as possible. Look to body nutrition. He also quoted cases of primary tubercular laryngitis from infection; greatest benefit from iodoform and morphia by careful insufflation; has seen cases apparently cured. The medium or tissue in which the bacilli thrive is to him more important than the bacilli themselves; do not see parasites in vigorous tissues. Those well-nourished are not liable to infection, not local treatment alone, but improve digestion and assimilation.

Dr. J. Solis-Cohen, of Philadelphia: Sulphurous treatment improves tissues or soil, so that germs cannot flourish as well; only recalls two cases of recovery from supposed tubercular laryngitis; scraped and applied lactic acid, also used morphia, iodoform and atropia, and believed in them; condemns tracheotomy to obtain rest.

Dr. E. Fletcher Ingals, of Chicago, finds it difficult to choose between high and low altitude. High altitudes are injurious where the disease is advanced. Calcium salts are beneficial; so, also, are the chlorides. The use of sulphuretted hydrogen, in his experience, increases pain and other unpleasant symptoms, so he abandoned it. He also had poor results from lactic acid. Morphia, carbolic acid and glycerole of tannin, with water, form excellent wash or spray. Tannin aids in alleviating pain by hardening the tissues. Glycerole of tannin without water is too irritating.

Dr. Coomes, of Louisville, Ky., finds that iodoform works best, and uses it extensively for the pain by insufflation after cleansing with Dobell's solution. Whisky and water is excellent to cleanse away the mucus and ease pain so as to allow patient to eat; had a bad case where he cured larynx completely, but patient died later from pulmonary deposits; has seen a case recover, even when the epiglottis had sloughed away.

Dr. Devilbiss, of Toledo, O., used both morphia and iodoform, but with vaseline and sprayed in; considers the liquid forms of vaseline the very best medium. He uses it with iodine and iodide of potassium.

Dr. Casselberry, of Chicago, doubts the good effect of lactic acid, as it causes too much irritation, increases the cough and does harm. Main point is to cleanse thoroughly, and use morphia and iodoform, or, better yet, iodol, which is lighter and more easily diffused. Temporary effect of cocaine is excellent, but continued use is bad. It causes reaction with congestion, increased irritation and inflammation. Morphia better, and with iodoform effect is for a longer time.

Dr. F. O. Stockton, of Chicago, Ill., has not had any success from scraping ulcers, and uses lactic acid from ten to ninety per cent. He saw a case in Vienna where there was both tubercular and syphilitic ulcerations of the larynx.

Dr. John Mackenzie, of Baltimore, is skeptical as to permanent cures, and thinks that they are more likely diphtheritic cases. He also doubts as to bacilli, with reference to cause and effect. Why will not they be caught in the nose of same individual when abraded surface? Is in favor of constitutional treatment; local treatment of bichloride, 1 to 2,000.

Dr. Curtis, of Chicago, says that atropine is of no use. Sprays the larynx with nitrate of silver, with the result of healing the ulcers.

Dr. Browne closed the discussion. He believes in the bacillary origin of the disease, but treats both bacillus and tissue as well. Note the close relation of bacillus of tuberculosis to that of lupus; must treat individual cases differently. He has had no good results from large doses of quinine, as it was not assimilated. Iodide of potash proved harmful, by causing loss of weight and interfering with nutrition. It is of benefit only in syphilitic laryngitis, which can readily be mistaken for tubercular.

